# Renal glucosuria is associated with lower body weight and lower rates of elevated systolic blood pressure: results of a nationwide cross-sectional study of 2.5 million adolescents

**DOI:** 10.1186/s12933-019-0929-7

**Published:** 2019-09-25

**Authors:** Boris Fishman, Gadi Shlomai, Gilad Twig, Estela Derazne, Alexander Tenenbaum, Enrique Z. Fisman, Adi Leiba, Ehud Grossman

**Affiliations:** 1 Israel Defense Forces, Medical Corps, Tel Hashomer, Ramat Gan, Israel; 2 Internal Medicine D and Hypertension Unit, Sheba Medical Center, 2 Derech Sheba, Migdal Ishpuz, 1st Floor, Tel Hashomer, 5265601 Ramat Gan, Israel; 3 Sackler Faculty of Medicine, Tel Aviv University, 6997801 Tel Aviv, Israel; 4 The Institute of Endocrinology, Sheba Medical Center, Tel Hashomer, 5265601 Ramat Gan, Israel; 5 Department of Military Medicine, The Hebrew University of Jerusalem, Jerusalem, Israel; 6 Cardiac Rehabilitation Institute, Sheba Medical Center, Tel Hashomer, 5265601 Ramat Gan, Israel; 7 Division of Nephrology and Hypertension, Assuta Ashdod Academic Medical Center, 7747629 Ashdod, Israel; 8 Faculty of Health sciences, Ben Gurion University, Beer Sheva, Israel; 9 Department of Medicine, Mount Auburn Hospital, 330 Mt Auburn St, Cambridge, MA 02138 USA; 10 Department of Medicine, Harvard Medical School, Boston, USA

**Keywords:** Glucosuria, Obesity, Overweight, Blood pressure

## Abstract

**Background:**

Gene coding mutations found in sodium glucose co-transporters (SGLTs) are known to cause renal glucosuria. SGLT2 inhibitors have recently been shown to be effective hypoglycemic agents as well as possessing cardiovascular and renal protective properties. These beneficial effects have to some extent, been attributed to weight loss and reduced blood pressure. The aim of the current study was to evaluate the prevalence of renal glucosuria amongst a large cohort of Israeli adolescents and to investigate whether renal glucosuria is associated with lower body weight and lower blood pressure values.

**Methods:**

Medical and socio-demographic data were collected from the Israeli Defense Force’s conscription center’s database. A cross-sectional study to evaluate the association between conscripts diagnosed as overweight [BMI percentiles of ≥ 85 and < 95 and obesity (≥ 95 BMI percentile)] and afflicted with renal glucosuria was conducted. In addition, we assessed the association of renal glucosuria with elevated diastolic and systolic blood pressure. Multinomial regression models were used.

**Results:**

The final study cohort comprised 2,506,830 conscripts of whom 1108 (0.044%) were diagnosed with renal glucosuria, unrelated to diabetes mellitus, with males twice as affected compared to females. The adjusted odds ratio for overweight and obesity was 0.66 (95% CI 0.50–0.87) and 0.62 (95% CI 0.43–0.88), respectively. Adolescents afflicted with renal glucosuria were also less likely to have an elevated systolic blood pressure of 130–139 mmHg with an adjusted odds ratio of 0.74 (95% CI 0.60–0.90).

**Conclusions:**

Renal glucosuria is associated with lower body weight and obesity as well as with lower rates of elevated systolic blood pressure.

## Background

Kidneys play a major role in glucose homeostasis. Up to 50% of fasting gluconeogenesis may be attributed to the kidney cortex [1, 2]. In a normal state, the kidneys reabsorb all of the glucose filtered by the glomeruli (~ 180 g/day). Elevated blood glucose levels reaching the kidneys’ maximum reabsorption capacity, result in glucosuria. The process of glucose reabsorption occurs in the proximal tubules of the nephron by transmembrane transporters of two gene families: the glucose transporters (GLUTs) and the sodium coupled glucose transporters (SGLTs) [3, 4]. The former and specifically, the GLUT2 and GLUT1 found in the proximal tubule epithelial cells, are a group of transporters located on the basolateral membrane, facilitating the passive transport of glucose back into the circulation [5, 6]. The SGLT2, located on the apical side, is a transmembrane transporter responsible for > 90% of the glucose reabsorption from the glomerular filtrate, whereas, the SGLT1 reabsorbs the remaining 10% [4]. There are at least 50 autosomal dominant and autosomal recessive mutations in the SGLT2 coding gene (SLC5A2), underlying familial renal glucosuria (FRG) [7, 8]. Although FRG may cause glucosuria of > 100 g/day, the affected individuals are asymptomatic without an increased risk of diabetes mellitus (DM) or urinary tract infections (UTIs) [2, 8].

The benign nature of FRG, as well as the nearly exclusive expression of SGLT2 in the kidney, has rendered the SGLT2s an intriguing pharmacological target in the development of novel hypoglycemic medications [1, 9]. Subsequently, over the past few years, several SGLT2 inhibitors have been approved by the US Food and Drug Administration for treating patients with type 2 diabetes [10]. Empagliflozin, dapagliflozin and canagliflozin have been shown to be effective oral hypoglycemic agents, but more importantly, these agents were found to be beneficial in reducing cardiovascular morbidity and mortality as well as the progression of diabetic renal diseases [11–14]. One of several proposed mechanisms underlying these positive dramatic effects on cardiovascular-related outcomes are weight reduction and blood pressure (BP) lowering effects of the SGLT2 inhibitors observed in pre-clinical and clinical trials [11–13, 15].

The aim of our study was to illustrate in a large population-based cohort of Israeli adolescents, the prevalence of non-diabetic renal glucosuria (RG) and investigate whether these subjects were less likely to exhibit cardiovascular risk factors such as increased body weight and elevated BP levels.

## Methods

### Databases and study population

All male and female Israeli adolescents, eligible for mandatory military service in the Israeli Defense Forces (IDF), are obligated to undergo a medical health assessment approximately 1 year prior to conscription. The medical assessment for military service candidates comprises a detailed medical history based on a status report completed by the candidates’ primary care physician and a thorough physical examination. Basic ancillary tests also include height, weight, BP measurements and a dipstick urinalysis. Valid computerized data relating to RG have been available since 1974. A glucosuria diagnosis is based upon a positive repeated qualitative urine dipstick prompting an evaluation to rule out DM and kidney disease which includes a morning fasting glucose, a 2-h oral glucose tolerance test and a biochemistry evaluation including serum creatinine and electrolytes. In the event of any missing data or abnormal findings during the initial assessment, the examinees were sent for a further, more comprehensive medical evaluation. All confirmed diagnoses were numerically coded by a trained military medical board.

Complete valid computerized data regarding BP measurements have been available since 1977. BP was measured in a sitting position, after a 5–10 min rest period, by a manual sphygmomanometer with an appropriately-sized cuff placed on the right arm, at heart level. Data as to socio-demographic characteristics were collected through personal interviews with all examinees. Our study included subjects aged 16–19 years, examined between January 1, 1974 and December 31, 2016. We excluded adolescents diagnosed with DM (n = 3113) and proteinuria (n = 4743) (Additional file 1: Figure S1). Computerized BP records of 6193 examinees examined after 1977 are nonexistent, therefore, they were excluded as well.

### Statistical analyses and variables

The IBM SPSS version 23 software (IBM SPSS; Somers, NY) was used for statistical analyses in our study. Univariate and multivariable logistic regression models evaluated the crude, adjusted odds ratio (aOR) and the 95% confidence interval (95 CI%) for RG, amongst males and females. The multivariable models were adjusted for year of examination, age at the time of examination, country of origin, body mass index (BMI), socio-economic status (SES) and years of education.

Univariate and multivariable multinomial regression models were conducted to evaluate the crude, the adjusted OR and the 95% CI, respectively, for weight status and systolic blood pressure (SBP) and separately for diastolic blood pressure (DBP). We adjusted the multivariable models for variables associated with the outcomes in the univariate analyses and for variables known to be associated with the outcome based on previous research. Several multivariable analyses were performed; the first, Model 2, was adjusted for the year of examination. Model 2A, (performed only for BP analyses) was adjusted for year of examination and BMI. Model 3, was also adjusted for sex, age at the time of examination, country of origin (i.e. their country of birth and in cases of Israeli-born, the country of origin was designated by their father’s country of origin). Model 4, in addition to the variables of the former models, was also adjusted for SES based on the examinee’s place of residence which was divided into three groups based on a scale taken from the Israeli Central Bureau of Statistics (low, middle and high).

Years of education were divided into four groups (9, 10, 11, 12 or more years of education); weight status into seven BMI percentile groups according to the Centers for Disease Control and Prevention Growth Charts [16]: < 5% (underweight), 5–24%, 25–49% (reference group), 50–74%, 75–84%, 85–94% (overweight) and ≥ 95% (obese). SBP was divided into five groups according to mmHg: SBP < 110, 110 ≤ SBP < 120 (reference group), 120 ≤ SBP < 130, 130 ≤ SBP < 140 and SBP ≥ 140; DBP was also divided into five groups according to mmHg: DBP < 70, 70 ≤ DBP < 80 (reference group), 80 ≤ DBP < 85, 85 ≤ DBP < 90 and DBP ≥ 90 mmHg.

Due to previous reports of possible increased risk UTIs and genital infections among patients treated with SGLT2 inhibitors [11, 17], we also looked for the association of RG and previous diagnoses of pyelonephritis or recurrent UTIs as reported by the examinees’ primary care physician. We did not have data regarding previous genital infections.

## Results

Detailed baseline characteristics of the study cohort are presented in Table 1. The final study cohort was comprised of 2,506,830 individuals of whom 1108 (0.044%) were diagnosed with RG unrelated to DM (Table 1); 74.6% participants diagnosed with RG were males, whereas, in the non-RG population, males comprised only 58.6% of the cohort (Table 1). The study cohort diagnosed with RG were from the same SES and had similar distributions of educational levels and ethnic origins compared to the non glucosuric participants (Table 1). RG was more prevalent in males (0.056%) than in females (0.027%) with a crude OR of 2.08 (95% CI 1.81–2.38) and an aOR of 1.96 (95% CI 1.70–2.25) (Table 1).

**Table 1:** Baseline characteristics of 2,506,830 16–19 year old participants examined during 1974–2016

Variable	Non glucosuric	Glucosuric
Number	2,505,722	1108
Males (% of all group)^a^	1,468,891 (58.6%)	827 (74.6%)
Age (mean ± SD)	17.3	17.6
Body mass index (kg/m^2^) [mean]	21.32	20.91
Systolic blood pressure (mmHg) [mean]	117	118
Diastolic blood pressure (mmHg) [mean]	71	72
Socio-economic status (% at each SES category)		
Low (1–4)	25.8	22.3
Moderate (5–7)	51.2	54.6
High (8–10)	21.7	22.2
Level of education (%)		
≤ 9 years	5.3	5.1
10 years	6	7.9
11 years	38.8	40
≥ 12 years	49.9	47
Country of origin (%)		
Israel	10	10.2
USSR	14.7	14.1
Asia	22.1	24.3
Africa	22.3	21.1
Europe + North America	26.4	23.1
Ethiopia	1.1	2.5
Minorities	1.9	3.8

*USSR* Union of Soviet Socialist Republics, *OR* odds ratio, *CI* confidence interval

^a^Crude OR for glucosuria amongst males compared to females was 2.08 (95% CI 1.81–2.38) and an adjusted OR of 1.96 (95% CI 1.70–2.25)

### Renal glucosuria and body mass index

**Fig. 1:**
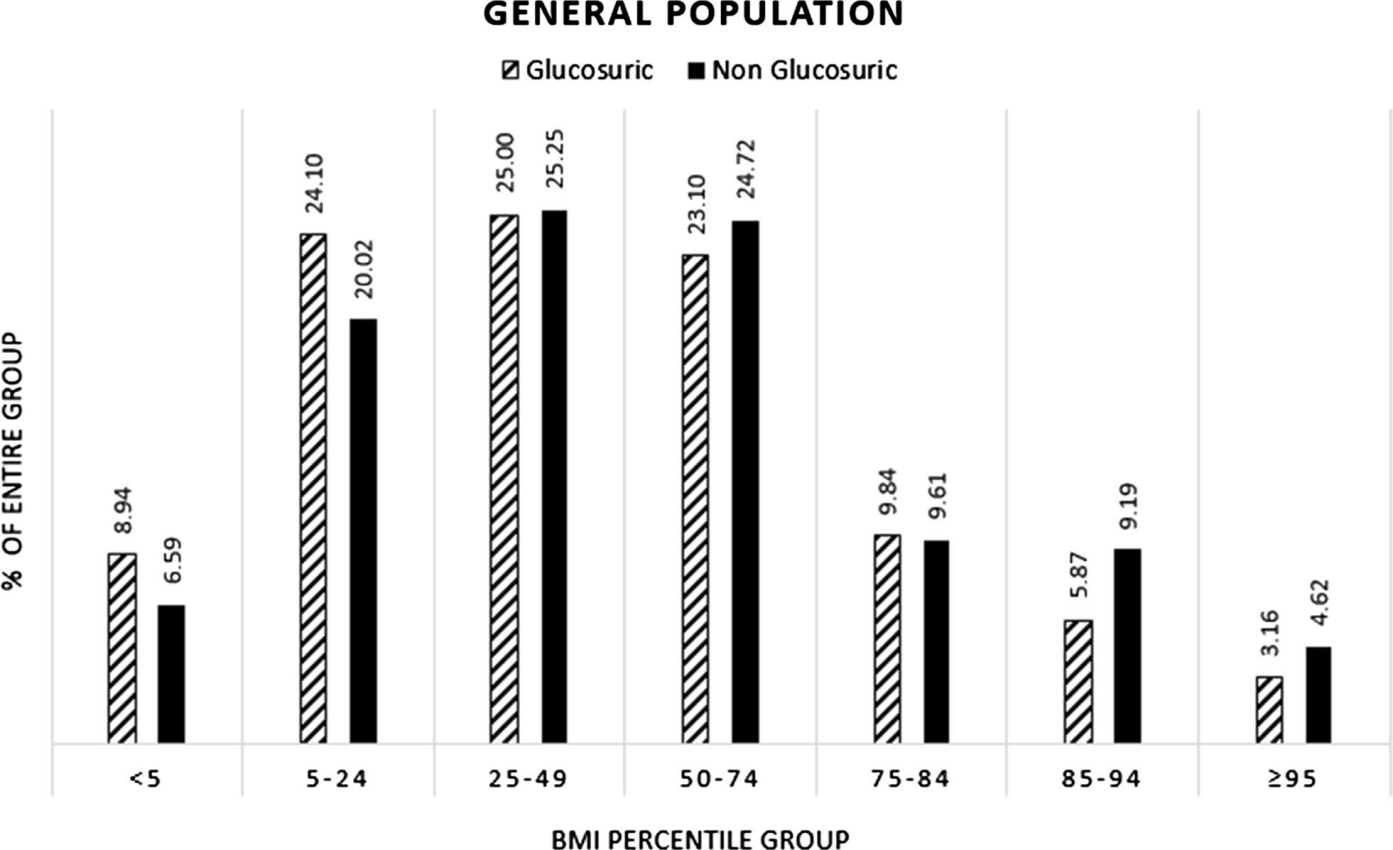
Body mass index percentile group distribution amongst the non glucosuric adolescents compared to glucosuric adolescents medically examined during the years of 1974–2016. General population. (N = 2,506,830)

Mean BMI was 20.91 kg/m^2^ in the RG group and 21.32 kg/m^2^ in the non-glucosuric group (p < 0.001). BMI percentile distribution in those diagnosed with RG and those without RG are shown in Fig. 1. Compared to the reference group (25–49 BMI percentile) in the univariate nominal regression for the general cohort population, the crude OR was 1.37 (95% CI 1.09–1.72), 0.64 (95% CI 0.49–0.84) and 0.69 (95% CI 0.49–0.98) for underweight, overweight and obesity, respectively (Table 2). In the final adjusted model, the aOR was 1.12 (95% CI 0.89–1.42), 0.66 (95% CI 0.50–0.87) and 0.62 (95% CI 0.43–0.88), for underweight, overweight and obesity, respectively (Table 2). A subgroup analysis of male distribution of BMI percentiles amongst those diagnosed with and without RG is shown in Additional file 1: Figure S2. The aOR in the final model was 1.14 (95% CI 0.88–1.49), 0.71 (95% CI 0.52–0.98) and 0.61 (95% CI 0.41–0.92) for underweight, overweight and obese males, respectively (Additional file 1: Table S1).

**Table 2:** Glucosuria and BMI percentiles in the general population, 1974–2016 (N = 2,506,830)

BMI percentile/model	< 5		5–24		50–74		75–84		85–94		≥ 95	
	OR	CI 95%	OR	CI 95%	OR	CI 95%	OR	CI 95%	OR	CI 95%	OR	CI 95%
Model 1	1.37	1.09–1.72	1.22	1.03–1.44	0.94	0.80–1.12	1.03	0.83–1.29	0.64	0.49–0.84	0.69	0.49–0.98
Model 2	1.37	1.09–1.72	1.22	1.03–1.44	0.94	0.79–1.11	1.01	0.81–1.27	0.63	0.48–0.82	0.65	0.46–0.93
Model 3	1.01	0.87–1.40	1.13	0.96–1.34	0.98	0.83–1.16	1.09	0.87–1.36	0.65	0.49–0.85	0.60	0.42–0.86
Model 4	1.12	0.89–1.42	1.15	0.97–1.36	0.99	0.83–1.17	1.11	0.89–1.38	0.66	0.50–0.87	0.62	0.43–0.88

The results of multinomial regression models

Five BMI percentiles (underweight), 5–24%, 25–49% (indicator group), 50–74%, 75–84%, 85–94% (overweight) and > 95% (obese). Reference group: 25 ≤ BMI < 50

Model 1 represents the crude odds ratios. Model 2 is adjusted for year (of examination at the conscription center). Model 3 is adjusted for year, age (at the time of the examination), sex and country of origin (grouped: Israel, USSR, Asia, Africa, Europe and North America, Ethiopia and minorities). Model 4 is adjusted for year, age, sex, country of origin, education status 9, 10, 11 and 12 or more years of education) and socio-economic status (divided into three groups according to the Israeli Central Bureau of Statistics

### Renal glucosuria and blood pressure

The relationship between RG and BP was assessed in 2,374,157 examinees (1,384,360 males [58.3%]) of whom 1058 [786 males (74.3%)] had been diagnosed with RG. The mean BP was 118/72 mmHg in the RG group and 117/71 mmHg in the non-glucosuric group (p = 0.10 and p = 0.14 for DBP and SBP, respectively). Compared to a reference of SBP 110–119 mm/Hg in the final adjusted model, the aOR was 0.74 (95% CI 0.60–0.90) for SBP of 130–139 mm/Hg (Table 3, Additional file 1: Figure S3). In a subgroup analysis of males, the aOR in the final model was 0.74 (95% CI 0.59–0.92) (Additional file 1: Table S2, Figure S4). No statistically significant differences were observed between the RG and non-RG subjects as to DPB values, including a subgroup analysis of males only (Table 3, Additional file 1: Figure S2, respectively). Of note, the glucosuric examinees did not have a higher rate of recurrent UTI (0.05% for both, data not shown).

**Table 3:** Glucosuria and blood pressure of the general population, 1977–2016 (N = 2,374,157)

BP	Systolic blood pressure								Diastolic blood pressure							
	SBP < 110		120 ≤ SBP < 130		130 ≤ SBP < 140		SBP ≥ 140		DBP < 70		80 ≤ DBP < 85		85 ≤ DBP < 90		DBP ≥ 90	
	OR	CI 95%	OR	CI 95%	OR	CI 95%	OR	CI 95%	OR	CI 95%	OR	CI 95%	OR	CI 95%	OR	CI 95%
Model 1	0.87	0.73–1.05	1.13	0.97–1.32	0.86	0.70–1.04	1.00	0.74–1.35	0.91	0.79–1.06	1.00	0.86–1.16	1.10	0.77–1.56	1.31	0.89–1.93
Model 2	0.87	0.72–1.04	1.14	0.98–1.33	0.85	0.70–1.04	1.02	0.76–1.38	0.88	0.76–1.03	1.03	0.88–1.20	1.08	0.76–1.54	1.34	0.91–1.98
Model 2A	0.84	0.70–1.01	1.17	1.01–1.37	0.91	0.75–1.11	1.14	0.84–1.54	0.86	0.74–1.00	1.06	0.91–1.23	1.15	0.81–1.64	1.46	0.99–2.15
Model 3	0.96	0.80–1.16	1.05	0.90–1.22	0.73	0.59–0.89	0.84	0.62–1.14	0.89	0.77–1.04	0.98	0.84–1.14	0.99	0.69–1.42	1.17	0.79–1.75
Model 4	0.97	0.80–1.17	1.06	0.91–1.24	0.74	0.60–0.90	0.86	0.63–1.17	0.90	0.77–1.05	0.99	0.85–1.15	1.02	0.71–1.46	1.20	0.81–1.79

The results of multinomial regression models

Reference groups: 110 ≤ SBP < 120, 70 ≤ DBP < 80 Model 1 represents the crude odds ratios. Model 2 is adjusted for year (of examination in the conscription center). Model 2A is adjusted for year, BMI (divided into seven groups by CDC percentiles). Model 3 is adjusted for year, (divided for 7 percentile groups), age (at the time of the examination), sex and country of origin (grouped for: Israel, USSR, Asia, Africa, Europe and North America, Ethiopia and minorities). Model 4 is adjusted for year, (divided into 7 percentile groups), age, sex, country of origin, education status 9, 10, 11 and 12 or more years of education) and socio-economic status (divided into three groups according to the Israeli Central Bureau of Statistics scale)

SBP and DBP measured in mm/Hg

*BP* blood pressure, *OR* odds ratio, *CI* confidence interval, *SBP* systolic blood pressure, *DBP* diastolic blood pressure

There were overall 479 and 2088 cases of pyelonephritis and recurrent UTIs, respectively, in our cohort. There were no cases of pyelonephritis in the glucosuric group and only one case of recurrent UTIs in the glucosuric group.

## Discussion

This nationwide cross-sectional study comprising 2,506,830 individuals is the largest study to date describing the prevalence of non-DM associated RG amongst adolescents. The prevalence of RG in our entire cohort was 0.044%, whereas, RG was more common amongst males, with a nearly twofold increase in the adjusted risk for RG compared to females. Our findings slightly differ from the results of two recent Southeast Asian studies, where the rates of RG were 0.05–0.07%, with similar rates appearing amongst males and females [18, 19]. This difference may stem from the genetic variability of the Southeast Asian population compared to the Israeli subjects. Furthermore, we showed that glucosuric adolescents were less likely to be overweight, obese or exhibit an elevated systolic BP.

Renal glucosuria is an uncommon, albeit well-recognized medical condition, with a benign clinical course [5, 7]. Over the past three decades, enormous progress has been made in discovering the underlying mechanisms for glucose reabsorption from the ultra-filtrate in the proximal tubules of the nephron [6, 15]. Previous studies have demonstrated the mutations in the gene expressing SGLT2 transmembrane transporter responsible for most cases of RG [5, 7]. These inhibitors are beneficial in reducing cardiovascular morbidity, mortality and the progression of diabetic renal [11–13, 20, 21]. One of the proposed mechanisms underlying these cardiovascular beneficial effects is reduced weight and BP values [15]. Dapagliflozin was shown to reduce total body weight and fat, particularly in visceral and subcutaneous adipose tissue [22–24]. Furthermore, in patients with type 2 diabetes, canagliflozin treatment for 52 weeks generated a 4 kg reduction in weight, most attributable to fat tissue loss. Weight reduction effects remained significant even after 2 years of follow-up [25]. Similar effects on body weight reduction were also noted following empagliflozin treatment [24, 26]. Correspondingly, in our study, glucosuric participants in the general cohort, males in particular, were less likely to be overweight or obese. This effect is probably best explained by the caloric loss caused by vast glucose urination [10, 15].

This hypothesis is further supported by data showing that patients treated with dapagliflozin may lose up to 300 k/cal daily [27]. Moreover, treatment with empagliflozin [28] and dapagliflozin [29, 30] were both found to lower SBP and DBP. Furthermore, a recent large meta-analysis of 27 randomized clinical trials, including ~ 13,000 participants, revealed that treatment with several SGLT2 inhibitors was associated with a mean reduction in SBP and DBP of 4 mmHg and 1.9 mmHg, respectively, without significant postural adverse effects [31].

In our study, we found that glucosuric subjects were less likely to experience an increased SBP in the 130–139 mmHg range. There was no significant protective effect when SBP was > 139 mm/Hg, probably due to the low statistical power, since there were only < 50 glucosuric adolescents with a SBP of > 139 mmHg. The BP reduction may be attributed to an osmotic diuresis effect as evidenced by the large amounts of glucose and sodium measured in the patient’s urine treated with these agents [10, 31–33].

It is noteworthy that all subjects in our study were examined to exclude DM and despite their normal blood glucose levels, they were less likely to be overweight or obese. Our results strengthen the assumption that most of the weight loss and BP reduction effects of the SGLT2 inhibitors are mediated through the glucosuria. This effect was manifested in all age groups, in non-diabetics and probably even more so in diabetic subjects whose potential loss of glucose is more significant and therefore, may have more significant effects on BMI and BP. A recent study of a Japanese middle-aged and elderly population found that glucosuric subjects (without a diagnosis of DM) had a higher mean BMI, SBP and DPB compared to non-glucosuric subjects [34]. However, the glucosuric group had a much higher mean creatinine level and higher rate of proteinuria and hematuria. Therefore, the glucosuria in this population was almost certainly the result of kidney disease in an older population. Contrary to this, our glucosuric subjects were adolescents and their glucosuria was probably due to a congenital abnormality rather than an acquired abnormality related to kidney disease. Our data raise the possibility of a beneficial effect of SGLT2 inhibitors in patients with prediabetes, mainly, those with a metabolic disease in order to prevent a progression to diabetes and a reduced cardiovascular risk. It is essential for future studies to prove this claim.

Our study has several notable limitations. Firstly, we had no data as to the severity of glucosuria amongst examinees diagnosed with RG. Secondly, study participants were analyzed as a homogenous group regardless of the specific genetic mutation associated with RG. Theoretically, different mutations may be associated with varying degrees of glucosuria, thereby, inducing variable body weight and BP reductions. Furthermore, our glucosuria estimation was qualitative and not quantitative, hence, we could not draw any conclusions as to the effects of increasing urine glucose and any measured outcomes. Thirdly, the BP values analyzed in our study were derived from a single BP office measurement taken in a potentially stressful environment (known to increase the probability of white coat-associated elevated BP measurement) [35]. Yet, most previous studies evaluating hypertension amongst adolescents, based their diagnosis upon only one BP measurement. Moreover, all examinees were uniformly exposed to the same stressful environment and therefore, the difference in BP values between subjects diagnosed with and without RG cannot be related to the stress.

## Conclusions

To the best of our knowledge, the current study is the largest trial to date evaluating the prevalence of RG in a late-adolescent population without DM. Our data indicate that in adolescents, RG is more common in males and is significantly associated with lower body weight, less obesity and lower rates of SBP values in the hypertension range. These findings may support the assumption that SGLT2 inhibitors may reduce weight and BP in non-diabetic individuals. Well-powered large scale studies are essential in order to clarify whether these RG-associated beneficial metabolic effects in adolescence have any ramifications on future cardiovascular morbidity and mortality. Well-powered large scale studies are essential in order to clarify whether these RG-associated beneficial metabolic effects in adolescence have any ramifications on future cardiovascular morbidity and mortality rates.

## Supplementary information

**Additional file 1: Figure S1.** Flow chart describing the study cohort (1974–2016). **Figure S2.** BMI percentile group distribution amongst males N = 1,469,718 (1974–2016). **Figure S3.** Systolic blood pressure groups distribution amongst the general population N = 2,374,157 (1977–2016). **Figure S4.** Systolic blood pressure group distribution amongst males N = 1,384,360 (1977–2016). **Table S1.** Glucosuria and BMI percentiles in males only (1974–2016). N = 1,469,718. Results of multinomial regression models. **Table S2.** Glucosuria and blood pressure in males only. (1977–2016). N = 1,384,360. Results of multinomial regression models.

## Data Availability

The databases used in our study were based on Israeli Defense Forces registries and are stored on Israeli Defense Forces computers. These computers are connected solely to the military network. These databases cannot be transferred to other computers or shared on the web, due to Israeli Defense Forces data security restrictions.

## Abbreviations

SGLTs: sodium glucose co-transporters
BMI: body mass index
CI: confidence interval
GLUTs: glucose transporters
FRG: familial renal glucosuria
DM: diabetes mellitus
UTIs: urinary tract infections
BP: blood pressure
RG: renal glucosuria
IDF: Israeli Defense Forces
SES: socio-economic status
SBP: systolic blood pressure
DBP: diastolic blood pressure
OR: odd ratios
aOR: adjusted odd ratios
