# Supplementary material for: Renal glucosuria is associated with lower body weight and lower rates of elevated systolic blood pressure: results of a nationwide cross-sectional study of 2.5 million adolescents
Source: Cardiovasc Diabetol. 2019 Sep 25;18:124. doi: 10.1186/s12933-019-0929-7 (PMC6760097; doi:10.1186/s12933-019-0929-7)
Supplement: Supplementary file 1 — Additional file 1: Figure S1. Flow chart describing the study cohort (1974–2016). Figure S2. BMI percentile group distribution amongst males N = 1,469,718 (1974–2016). Figure S3. Systolic blood pressure groups distribution amongst the general population N = 2,374,157 (1977–2016). Figure S4. Systolic blood pressure group distribution amongst males N = 1,384,360 (1977–2016). Table S1. Glucosuria and BMI percentiles in males only (1974–2016). N = 1,469,718. Results of multinomial regression models. Table S2. Glucosuria and blood pressure in males only. (1977–2016). N = 1,384,360. Results of multinomial regression models. [file 12933_2019_929_MOESM1_ESM.docx]

**Figure S1: Flow chart describing the study cohort (1974-2016)**

**
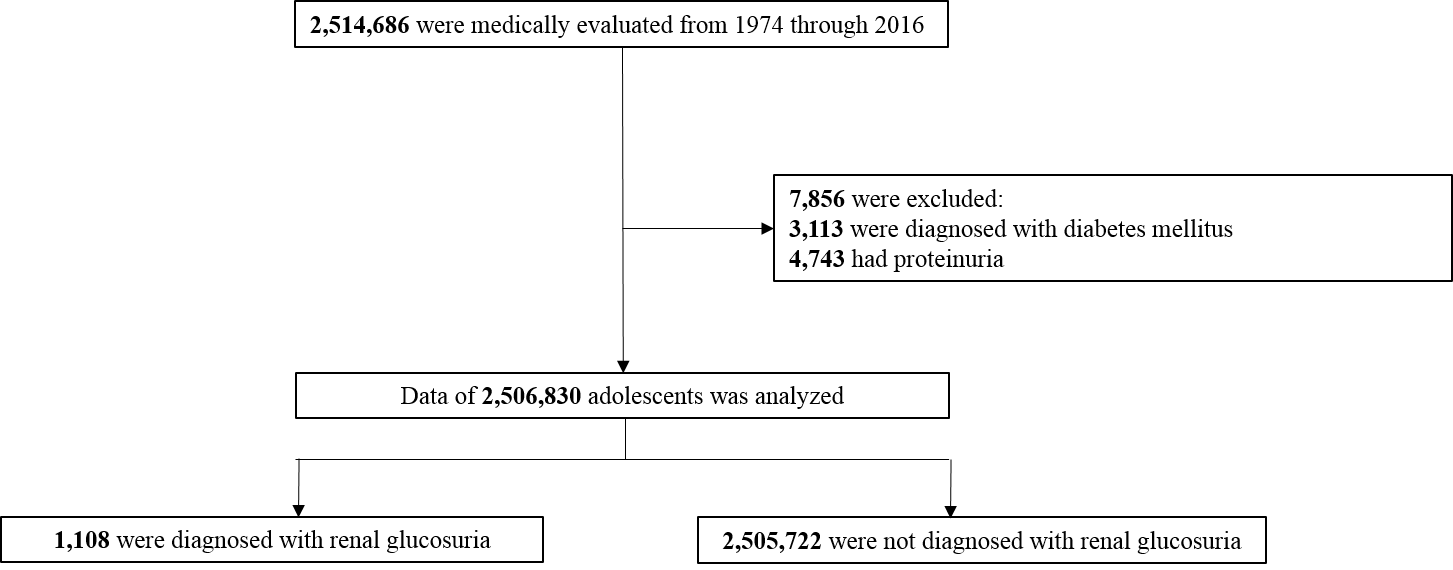
**

**Figure S2: BMI percentile group distribution amongst males N=1,469,718 (1974-2016)**


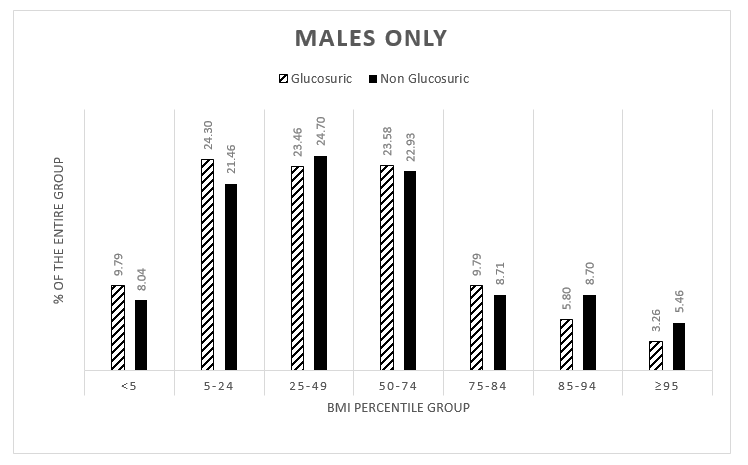


BMI- body mass index

**Figure S3: Systolic blood pressure groups distribution amongst the general population N=2,374,157 (1977-2016)**


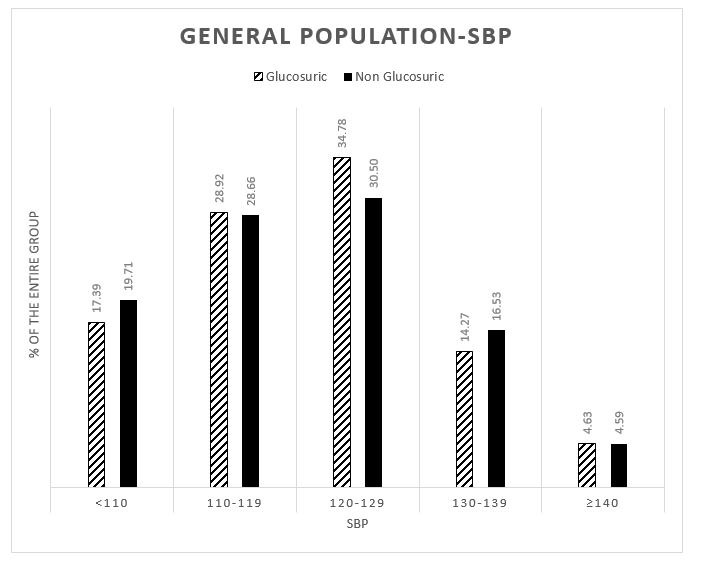


**Figure S4: Systolic blood pressure group distribution amongst males N=1,384,360 (1977-2016).**


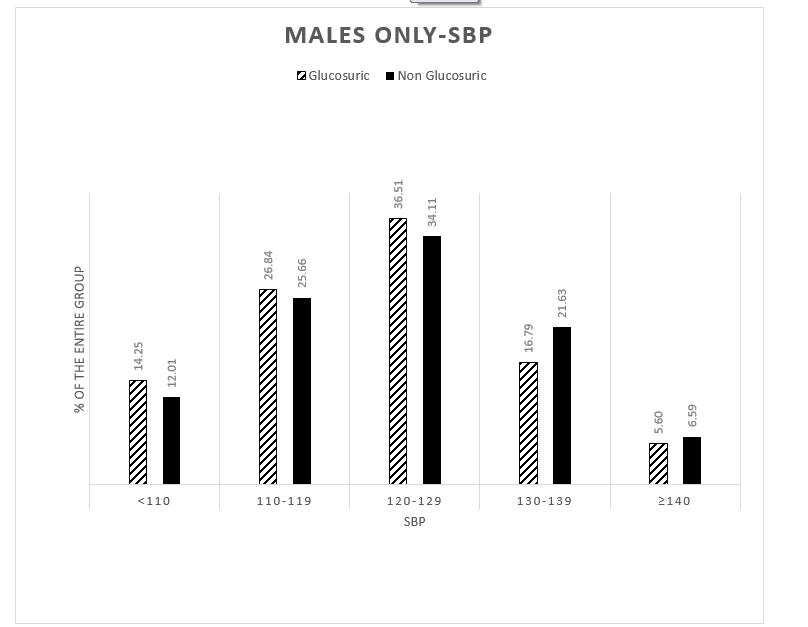


**Table S1: Glucosuria and BMI percentiles in males only (1974-2016). N=1,469,718. Results of multinomial regression models.**

| **BMI percentile/ model** | **<5** | | **5-24** | | **50-74** | | **75-84** | | **85-94** | | **≥95** | |
| --- | --- | --- | --- | --- | --- | --- | --- | --- | --- | --- | --- | --- |
|  | **OR** | **CI 95%** | **OR** | **CI 95%** | **OR** | **CI 95%** | **OR** | **CI 95%** | **OR** | **CI 95%** | **OR** | **CI 95%** |
| **Model 1** | 1.28 | 0.99-1.66 | 1.19 | 0.98-1.45 | 1.08 | 0.89-1.32 | 1.18 | 0.91-1.53 | 0.70 | 0.51-0.96 | 0.53 | 0.42-0.94 |
| **Model 2** | 1.28 | 0.99-1.66 | 1.20 | 0.98-1.46 | 1.07 | 0.88-1.31 | 1.16 | 0.89-1.50 | 0.68 | 0.49-0.93 | 0.60 | 0.40-0.90 |
| **Model 3** | 1.13 | 0.86-1.46 | 1.15 | 0.94-1.40 | 1.10 | 0.90-1.34 | 1.21 | 0.93-1.56 | 0.70 | 0.51-0.96 | 0.60 | 0.40-0.89 |
| **Model 4** | 1.14 | 0.88-1.49 | 1.16 | 0.95-1.42 | 1.10 | 0.90-1.34 | 1.22 | 0.94-1.59 | 0.71 | 0.52-0.98 | 0.61 | 0.41-0.92 |

**OR**-odds ratio, **CI**- confidence interval. **BMI**-body mass index. The BMI is the weight in kilograms divided by the square of the height.

Reference group: 25≤BMI<50 Model 1 represents the crude odds ratios. Model 2 is adjusted for year (of examination in the conscription center). Model 3 is adjusted for year, age (at the time of the examination), country of origin (grouped for: Israel, USSR, Asia, Africa, Europe and North America, Ethiopia and minorities). Model 4 is adjusted for year, age, country of origin, education status 9,10,11 and 12 or more years of education) and socio-economic status (divided into 3 groups according to the Israeli Central Bureau of Statistics scale).

**Table S2: Glucosuria and blood pressure –males only. (1977-2016). N=1,384,360. Results of multinomial regression models.**

|  | **Systolic Blood Pressure** | | | | | | | | | **Diastolic Blood Pressure** | | | | | | | | |
| --- | --- | --- | --- | --- | --- | --- | --- | --- | --- | --- | --- | --- | --- | --- | --- | --- | --- | --- |
| **Blood pressure** | **SBP < 110** | | **120≤SBP<130** | | **130≤SBP<140** | | **SBP≥140** | | | **DBP<70** | | **80≤DBP<85** | | **85≤DBP<90** | | **DBP≥90** | |  |
|  | OR | CI 95% | OR | CI 95% | OR | CI 95% | | OR | CI 95% | OR | CI 95% | OR | CI 95% | OR | CI 95% | OR | CI 95% |  |
| **Model 1** | 1.13 | 0.90-1.43 | l1.02 | 0.86-1.22 | 0.74 | 0.60-0.92 | | 0.81 | 0.59-1.12 | 0.99 | 0.83-1.18 | 1.01 | 0.85-1.20 | 1.21 | 0.83-1.76 | 0.95 | 0.58-1.54 |  |
| **Model 2** | 1.13 | 0.90-1.42 | 1.03 | 0.86-1.23 | 0.74 | 0.59-0.92 | | 0.83 | 0.60-1.14 | 0.96 | 0.80-1.15 | 1.04 | 0.87-1.24 | 1.21 | 0.83-1.75 | 0.97 | 0.6-1.58 |  |
| **Model 2A** | 1.11 | 0.88-1.39 | 1.05 | 0.88-1.26 | 0.78 | 0.63-0.97 | | 0.91 | 0.65-1.26 | 0.94 | 0.78-1.12 | 1.06 | 0.89-1.27 | 1.28 | 0.88-1.86 | 1.05 | 0.64-1.71 |  |
| **Model 3** | 1.13 | 0.90-1.42 | 1.03 | 0.86-1.23 | 0.73 | 0.59-0.91 | | 0.82 | 0.60-1.14 | 0.95 | 0.79-1.14 | 1.03 | 0.87-1.23 | 1.16 | 0.79-1.70 | 0.90 | 0.54-1.48 |  |
| **Model 4** | 1.12 | 0.89-1.42 | 1.04 | 0.87-1.24 | 0.74 | 0.59-0.92 | | 0.84 | 0.61-1.17 | 0.96 | 0.80-1.15 | 1.04 | 0.87-1.24 | 1.18 | 0.81-1.73 | 0.91 | 0.55-1.51 |  |

**OR**-odds ratio, **CI**- confidence interval. **SBP**-systolic blood pressure. **DBP**- diastolic blood pressure. SBP and DBP measured in mm/Hg.

Reference groups: 110≤SBP<120, 70≤DBP<80. Model 1 represents the crude odds ratios. Model 2 is adjusted for year (of examination in the conscription center). Model 2A is adjusted for year, BMI (divided into 7 groups by CDC percentiles). Model 3 is adjusted for year, (divided into 7 percentile groups), age (at the time of the examination), country of origin (grouped for: Israel, USSR, Asia, Africa, Europe and North America, Ethiopia and minorities). Model 4 is adjusted for year, (divided into 7 percentile groups), age, country of origin, education status 9,10,11 and 12 or more years of education) and socio-economic status (divided into 3 groups according to scale of Israeli central bureau of statistics).
